# Advances in Raman spectroscopy for characterising oral cancer and oral potentially malignant disorders

**DOI:** 10.1017/erm.2024.26

**Published:** 2024-10-08

**Authors:** Katie Hanna, Anna-Lena Asiedu, Thomas Theurer, David Muirhead, Valerie Speirs, Yara Oweis, Rasha Abu-Eid

**Affiliations:** 1 School of Medicine, Medical Sciences and Nutrition, University of Aberdeen, Scotland, UK; 2 Aberdeen Cancer Centre, University of Aberdeen, Scotland, UK; 3 School of Geoscience, University of Aberdeen, Aberdeen, Scotland, UK; 4 School of Dentistry, University of Jordan, Amman, Jordan

**Keywords:** detection, diagnosis, oral cancer, oral potentially malignant disorders, Raman spectroscopy

## Abstract

Oral cancer survival rates have seen little improvement over the past few decades. This is mainly due to late detection and a lack of reliable markers to predict disease progression in oral potentially malignant disorders (OPMDs). There is a need for highly specific and sensitive screening tools to enable early detection of malignant transformation. Biochemical alterations to tissues occur as an early response to pathological processes; manifesting as modifications to molecular structure, concentration or conformation. Raman spectroscopy is a powerful analytical technique that can probe these biochemical changes and can be exploited for the generation of novel disease-specific biomarkers. Therefore, Raman spectroscopy has the potential as an adjunct tool that can assist in the early diagnosis of oral cancer and the detection of disease progression in OPMDs. This review describes the use of Raman spectroscopy for the diagnosis of oral cancer and OPMDs based on ex vivo and liquid biopsies as well as in vivo applications that show the potential of this powerful tool to progress from benchtop to chairside.

## Introduction

Oral cancers are malignant tumours that arise in the oral cavity, affecting the gingiva, tongue, buccal mucosa, labial mucosa, floor of the mouth and palate. The subsite heterogeneity of oral cancers varies in terms of global prevalence, risk factors and treatment response. Oral squamous cell carcinoma (OSCC), an aggressive neoplasm of the oral epithelium, represents more than 90% of all oral cancers (Ref. 1). Recent estimates have placed oral cancer as the 18^th^ most common malignancy in the world, with 377 713 new cases and 177 757 deaths reported in 2020 (Ref. 2). The incidence of oral cancer varies geographically, with the highest incidence reported in Southeast Asia (Ref. 3). Geographical variations are related to differences in risk factors, of which many can be attributed to certain habits, like betel (areca) nut chewing, tobacco (in all its forms), and alcohol abuse and poor diet, as well as viral infections (e.g. human papilloma virus).

Whilst oral cancers can develop de novo, many are preceded by oral potentially malignant disorders (OPMDs), an umbrella term for a range of oral mucosal disorders with an increased risk of malignant transformation (Ref. 4). The rate and potential for malignant transformation of OPMDs vary according to several different factors, including the type and site of the lesion and the presence of dysplastic changes, termed oral epithelial dysplasia (OED) which encompasses a spectrum of cellular and architectural alterations evident under histological examination.

**Figure 1.**
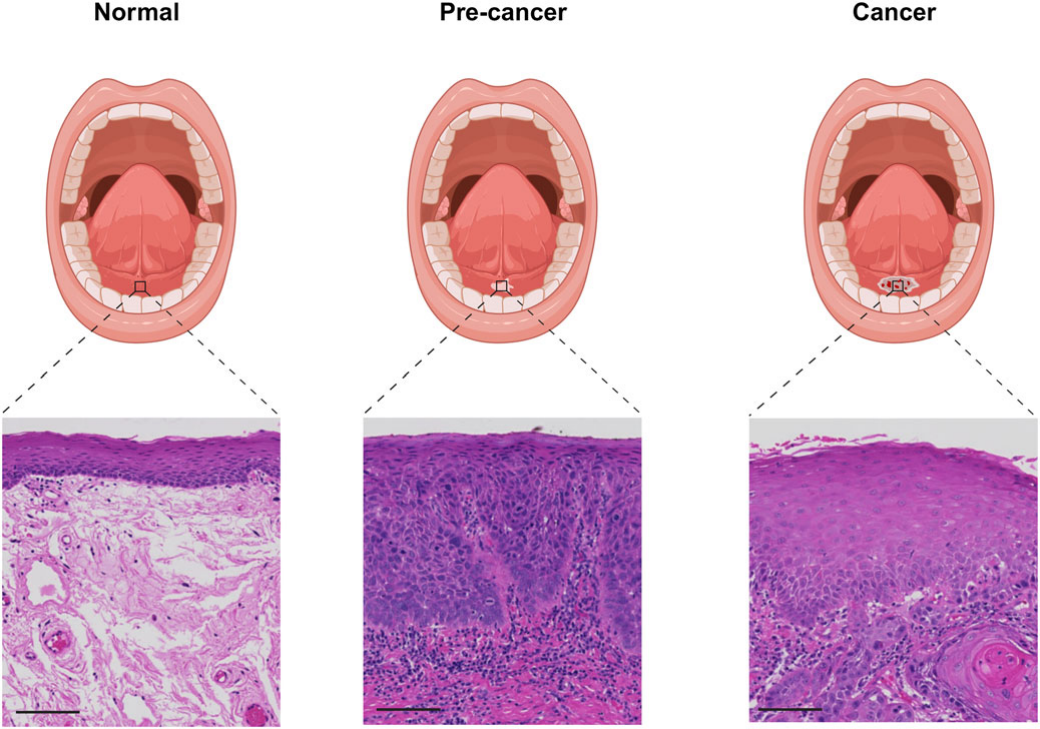
Schematic of anatomical presentation and histological representation of the progression of oral cancer in the mucosa lining the floor of the mouth. Histopathological images were obtained from NHS Grampian Biorepository as part of ongoing research projects (Approved by the Scientific Access Group (tissue requests TR000256 and TR000189), REC reference: 21/NS/0047, IRAS project ID: 296502). Haematoxylin and Eosin, 20x, scale bar represents 100 μm. Created in Biorender.

The current gold-standard for diagnosing oral cancer and OPMDs relies on intra-oral visual inspection clinically, followed by a biopsy and histopathological assessment to confirm the malignant or dysplastic changes. See Figure 1 for an illustration of the changes that accompany the progression from normal to premalignant to malignant oral epithelium. However, the current classification of dysplastic lesions is unreliable for detecting disease progression, thereby hindering early detection of malignant changes. Problems with the current classification systems arise from the subjectivity in evaluating diagnostic parameters of dysplasia in addition to the absence of validated criteria that are important for predicting malignant transformation (Ref. 5).

With the accessibility of the oral cavity, developing less invasive screening tools is possible. The application of optical technologies for the characterisation of oral cancers and OPMDs has been gaining popularity in recent years (Ref. 6). Alterations to normal tissue biochemistry are reflected in changes to its optical properties. Optical techniques are sensitive to biochemical changes that accompany malignancy, and as these changes often occur prior to morphological alterations, they can be used for screening and early diagnosis (Ref. 6).

Raman spectroscopy (RS) is one such optical approach that has been gaining significant momentum in the medical space, and some of its most significant applications are found within the field of oncology (Refs 7–11). First discovered in 1928 (Ref. 12), the Raman effect describes the phenomenon of inelastic photon scattering whereby incident (laser) photons interact with the electron cloud surrounding a molecular bond. This induces a localised dipole moment, generating a molecular vibrational state specific to the chemophysical nature of the bond. This vibrational state causes an energy shift in the photon as it is scattered following the interaction (Ref. 13). The difference in energy (∝ frequency) and wavelength between the incident and scattered photon, that is, the Raman shift, is proportional to discrete vibrational energies of a given molecular bond. As such, RS affords a means to produce a sample-specific fingerprint. The molecular specificity of the spectrum can be exploited for the generation of novel diagnostic, prognostic and predictive biomarkers.

**Figure 2.**
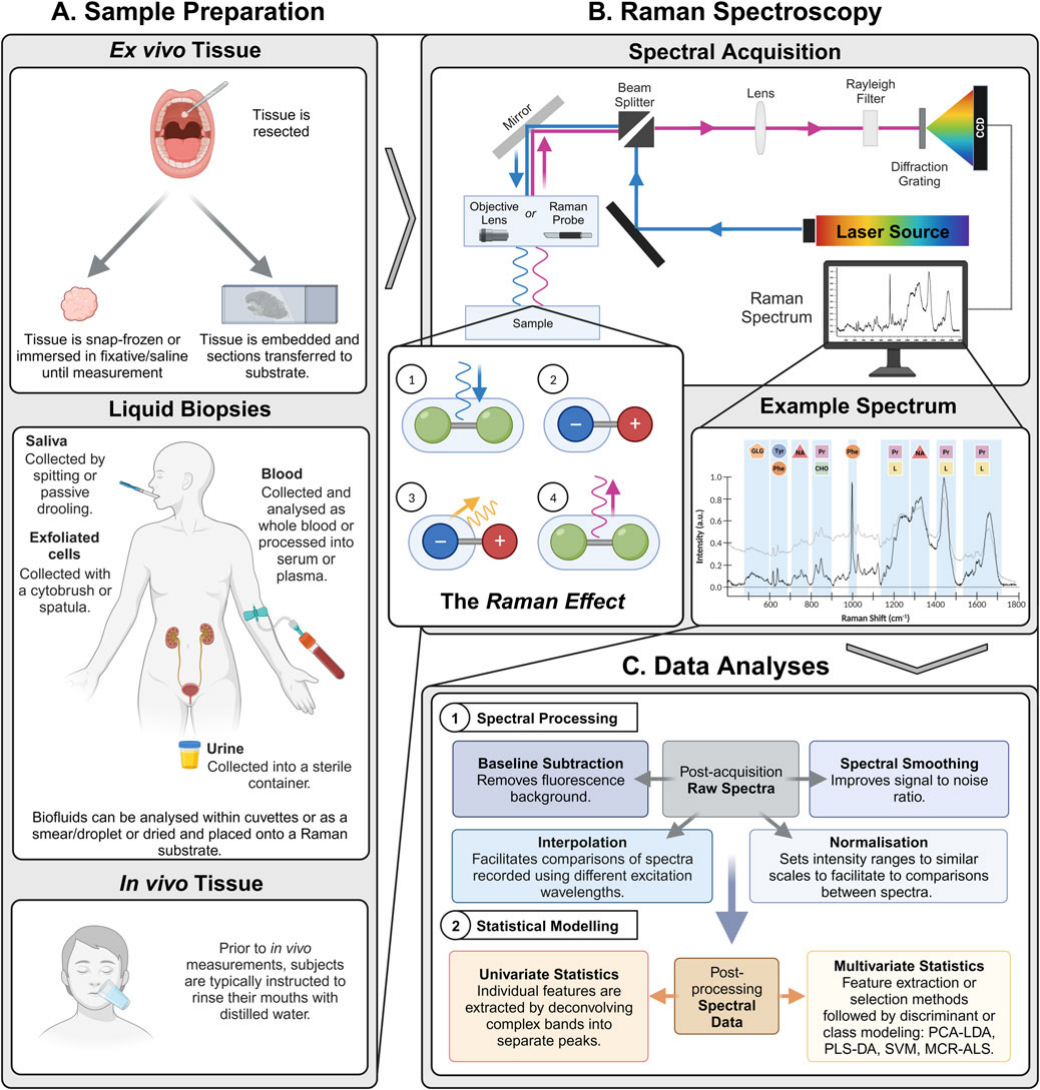
Typical workflow for the preparation of different types of biological sample for Raman spectroscopy. (A) Studies on the application of Raman spectroscopy for the detection and characterisation of oral cancer and OPMDs are primarily focused on the sampling of ex vivo tissue and liquid biopsies (saliva and exfoliated cells, blood (plasma and serum) and urine) or the oral mucosa, in vivo. (B) Following preparation, samples are illuminated with a laser beam focused through a microscope objective or probe onto the sample. As per ‘the Raman effect’, the excitation of the molecule during the photon interaction (1) alters the electron cloud (induced dipole moment) (2) and generates a vibrational mode, shown here as phonon release in yellow (3). The loss or gain of energy from the photon to generate the vibration causes the photon to be scattered at a different energy state (4). The scattered light is then collected through the collection optics. Rayleigh light is rejected through filters, but Raman-scattered light travels through these filters and is focused onto the dispersion grating that splits the beam into single wavelength components, which is then projected onto a charged coupled device detector. The detector converts the photons of light into an electrical signal and the Raman spectrum is generated and displayed on the computer monitor. The spectrum is a sample-specific fingerprint and is presented as the intensity of scattered light plotted against the Raman shift (the difference in frequency between the incident and scattered photon – expressed in wavenumbers (cm^−1^)). Raman active modes appear as bands at various frequencies, characteristic of structural features and functional groups of a particular molecule. The wavenumber of vibrational modes of biological samples are found predominantly within the biological fingerprint (400–1800 cm^−1^), and these include vibrations of glycogen (GLG), amino acids (tyrosine (Tyr), phenylalanine (Phe)), nucleic acids (NA), proteins (Pr), carbohydrates (CHO) and lipids (L). (C) Spectral pre-processing is critical for analysing Raman spectra, from biological samples, to remove variances arising from non-biological interferences, to ultimately enhance the biological signal. Pre-processing can include baseline correction, smoothing, interpolation and normalisation. The coupling of Raman spectroscopy with statistical approaches allows the transformation of highly complex data into manageable variables that confer the underlying biological mechanisms. Extracting features from Raman spectra and the subsequent generation of appropriate variables can be facilitated through univariate and multivariate approaches. Created in Biorender.

RS offers advantages for studying biological material: it does not require significant sample preparation or labelling; it is a versatile technique that can be employed for the study of cells, tissue and liquid biopsies and its non-destructive nature maintains the functionality and integrity of the biological sample under investigation. Figure 2 summarises the steps required to obtain Raman spectra from biological material, from sample harvesting and preparation through spectral acquisition to data pre-processing and analysis.

Several Raman variants have been developed including surface-enhanced Raman spectroscopy (SERS) and shifted-excitation Raman difference spectroscopy (SERDS) that seek to enhance the signal intensity of the weak Raman signal and to overcome strong autofluorescence interference, respectively. Extracting features from Raman spectra and the subsequent generation of appropriate variables can be through univariate and multivariate approaches. Full details of these Raman techniques and analysis methods are beyond the scope of this review, but the reader can refer to the Supplementary material for further information.

This review highlights the current applications of RS for the detection, diagnosis, characterisation and monitoring disease progression in oral cancers and OPMDs.

## Applications of Raman spectroscopy in oral cancer and OPMDs

RS has been used for characterising oral tumours and OPMDs. In this section, we will summarise the advances in RS use in ex vivo studies using solid and liquid biopsies and in vivo clinical applications.

### Ex vivo studies of oral tissues

The analysis of ex vivo tissue samples is the most widespread application of RS in oral cancer and OPMDs. These are mainly tissue sections analysed either as histological sections using microscopic Raman-based systems or small tissue samples using hand-held devices.

In this section, we will explore the applications of RS for the differentiation between oral healthy and cancerous tissues and for detecting changes across the spectrum of disease progression from normal through dysplastic to neoplastic oral tissues.

#### Raman spectroscopy in oral cancer and healthy tissue

The first applications of RS in oral cancer date back to the early 2000s (Ref. 14). This study showed a classification sensitivity and specificity greater than 85% for defining 12 normal and 37 malignant fresh oral tissue samples when Raman spectra were subject to principal component analysis (PCA). Normal spectra resembled those of lipids whilst malignant spectra resembled protein signatures (Ref. 14).

The same group later analysed formalin-fixed tissue sections from histo-pathologically confirmed malignant and normal oral biopsies. Whilst they probed both epithelial and sub-epithelial regions using Raman micro-spectroscopy, the latter regions did not afford reliable discrimination between healthy and cancer oral tissue. However, significant differences were observed between normal and malignant epithelial tissue regarding protein peaks between 850 and 1470 cm^−1^, attributed to both protein compositional and conformational changes (Ref. 15).

Spectra from normal and OSCC lingual tissue were analysed using a two-step PCA-hierarchical linear discriminant analysis (LDA) spectral classification model which demonstrated 91% accuracy, 100% sensitivity and 78% specificity (Ref. 16). Systematic analysis of the biochemical constituents from 1087 spectra from OSCC and healthy tissue structures in the tongue (surface squamous epithelium, muscle, adipose and connective tissue, glands and nerves) revealed that carbohydrates, proteins and amino acids could discriminate tumorous from healthy tissue with a sensitivity and specificity of 100 and 93%, respectively (Ref. 17). The relative intensities of the RS 1656 cm^−1^ band and the 1440/1452 cm^−1^ ratio could differentiate normal tissues from different OSCC grades (well-, moderately or poorly differentiated). Overall, these findings suggest a shift from lipid to protein signatures in malignancy (Ref. 18).

The reduction of lipid content in cancerous tissue may be a result of lipid peroxidation, which is a metabolic process where reactive oxygen species attack lipids containing C = C, leading to their deterioration. Oxidative stress plays a significant role in cancer metastasis and invasion (Ref. 19); therefore, the reduction of lipids in cancer cases could be linked to elevated levels of the reactive oxygen species (Ref. 20). Regarding the changes in proteins, in homeostatic conditions, protein synthesis is tightly regulated and determines proliferation rates, differentiation and stress adaptation (Ref. 21). When cells gain a tumour phenotype, this becomes deregulated, thus promoting aberrant cell division and metastasis, which could in turn lead to the observed higher levels of proteins.

The integrated areas of five normalised wavenumber regions – corresponding to phenylalanine (1004 cm^−1^), C–C stretch vibrations of the conjugated backbone (1156 cm^−1^), protein sidechains (1360 cm^−1^), C = C stretching of lipid or retinol (1587 cm^−1^) and the amide I protein vibration (1660 cm^−1^) – demonstrated capabilities for discriminating normal and cancerous oral mucosa when subject to PCA (Ref. 22). A spectral matching approach compared multivariate curve resolution and alternating least squares decomposed Raman spectra of tumour and normal tissue from OSCC patients to standard keratin Raman spectra. It was observed that tumour spectra were more similar to keratin, enabling OSCC detection with 77–92% sensitivity and 100% specificity (Ref. 23). Changes in the expression and distribution of cytokeratins are established molecular markers of OSCC (Ref. 24), which are currently identified by immunohistochemistry. RS could afford a simple, inexpensive and quick alternative for detection of changes in keratin in oral tissues.

A leaf-like silver titanium dioxide SERS substrate provided enhanced spectral signatures from cryo-sectioned oral tissue. A specificity and sensitivity of 95.83 and 100% were achieved for discriminating tumour and normal oral SERS spectra when coupled with PCA-DA. The reported turnaround time from spectral acquisition to classification was very short, thus demonstrating the translational potential of this method (Ref. 25). Furthermore, SERDS has been employed to analyse excised normal and OSCC tissue, where spectra were subject to PCA-LDA and cross-validation, revealing an 86.1 and 94.4% sensitivity and specificity, respectively (Ref. 26).

Normal and malignant Raman spectra were analysed using PCA-LDA and PCA-quadratic DA classifier models, subsequently validated by leave-one-out cross-validation (LOOCV) and k-fold cross-validation. The PCA-QDA outperformed PCA-LDA, with the former achieving a 90.9% sensitivity and 83.33% specificity and the latter achieving a 77.27 and 86.11% sensitivity and specificity, respectively. Moreover, that was the first study to use RS to perform a sub-site-wise differentiation of the lingual, buccal and gingival mucosae. Amide I (1655 cm^−1^) and Amide III (1250 cm^−1^) peaks were most prominent in buccal lesions, whereas protein/lipid bands at 1155 and 1523 cm^−1^ were more intense in the tongue and gingival sub-sites (Ref. 27). Malignancies of these sub-sites are known to differ in prognosis, metastasis to lymph nodes, aggressiveness and overall survival. The spectral differences could reflect biochemical changes that drive these different presentations, added to the physiological differences between these mucosal sites, which reflect their function as lining (buccal) or masticatory (tongue and gingiva) mucosae. When RS was combined with autofluorescence imaging using VELscope, there was a clear reduction in misclassification rates for OSCC detection, with the combination achieving 97.14% accuracy, 100% sensitivity and 94.3% specificity (Ref. 28).

Coupling RS with deep learning, using convolutional neural networks (CNN), has been employed previously to classify lingual OSCC and adjacent non-tumorous tissue. CNN, linear discriminate analysis and SVM were used to analyse Raman spectra to distinguish lingual OSCC and normal tissue. Combining CNN and SVM achieved 99.31% sensitivity and 94.44% specificity in detecting lingual OSCC (Ref. 29). A CNN model that identified lingual OSCC with a sensitivity of 99.07% and specificity of 95.37% was also found to have potential for delineating surgical margins (Ref. 30). Applying residual network (types of CNN) to build a diverse spectral band-based model could distinguish lingual OSCC from normal tissue with high sensitivity (97.38%) and specificity (98.75%) (Ref. 31). Utilising deep learning to analyse Raman spectra may help propel RS into the clinical setting as it has the potential to overcome the time-consuming spectral pre-processing and feature extraction for objective assessment of large datasets.

OSCC could be distinguished from surrounding healthy tissue based on differences in tissue water concentration, as determined by the signatures at higher wavenumbers (~>2000 cm^−1^) of freshly surgically excised tongue specimens. Overall, water concentration was observed to be significantly greater within the region of the tumour compared to that of the surrounding normal tissue (Ref. 32). Water concentration decreased from 76% within the tumour, to 59% within the inadequate surgical margins (<5 mm from tumour border), to 54% within the adequate surgical margins (>5 mm from tumour border) (Ref. 33). RS analysing water content could also discriminate healthy from diseased bone in bone resection margin assessment during OSCC surgery (Ref. 34). Changes in water concentration in different types of oral tissue, which can be quantified by RS, have the potential to be used for in vivo RS surgical margin demarcation.

Raman maps allow visualisation of the spatial distribution of biochemical species. Daniel *et al*. (2014) were the first to image oral tissue with RS, using frozen sections of normal and OSCC in the 1100–1700 cm^−1^ range. From this, pseudo-colour Raman maps were generated using cluster memberships obtained through PCA and k-means clustering. The normal samples exhibited 3–4 clusters whereas tumour samples showed 4–5 clusters. The mean spectrum from each cluster was analysed using PCA, which achieved a sensitivity, specificity and overall accuracy of 97.9, 99.14 and 98.9%, respectively, for distinguishing normal and cancerous tissue, respectively (Ref. 35). Raman maps from healthy and carcinogen-induced tissue from the buccal pouch of a hamster were constructed and, using PCA-LDA, could discriminate healthy from tumour spectra with high accuracy (Ref. 36).

#### Raman spectroscopy across the spectrum of disease progression

The capabilities of RS go beyond discriminating normal from malignant oral tissues and several studies have demonstrated its ability to distinguish subtle differences between various oral pathologies. Differences between the spectra of normal versus that of malignant, premalignant and inflammatory oral tissue were observed and PCA combined with multiparameter limit tests allowed for the discrimination of different tissue types. The most pronounced differences between normal and malignant spectra were observed within the 1200–1800 cm^−1^ region, whereas differences between pathological entities were most pronounced between 900 and 1400 cm^−1^ (Ref. 37). Moreover, in another study, peaks within 800–1800 cm^−1^ corresponding to lipids, as expected, became increasingly more intense from OSCC through premalignant (oral sub mucous fibrosis (OSMF) and leukoplakia) to normal tissue samples. Peaks corresponding to Amide I and III followed the opposite trend, suggesting a reduction in protein signatures in normal tissue compared to premalignant and malignant oral tissues. PC-LDA revealed a 97.4% accuracy for classifying the different groups (Ref. 33). An approach based on SERDS was tested to differentiate healthy mucosa, OSCC and non-malignant lesions (hyperkeratosis, inflammation, irritant fibroma, leukoplakia and dysplasia). The mean SERDS spectra showed noticeable differences between physiological and pathological tissues particularly between 1200 and 1800 cm^−1^, whereas differences between the different pathological states occurred predominately between 800 and 1400 cm^−1^ (Ref. 38). This observation was also made in an earlier study (Ref. 37), and suggests that this wavelength window of the spectrum should be investigated further for differentiating pathologies of the oral cavity. The spectra were subjected to PCA and then three separate classification models based on LDA were developed to investigate differentiation performance. The classification of healthy mucosa versus non-malignant lesions achieved an accuracy of 95.3%; that of healthy mucosa against OSCC an accuracy of 89.8%, and non-malignant lesions versus OSCC achieved an accuracy of 88.4% (Ref. 38).

RS was used to characterise tissue-engineered 3D models of normal, dysplastic and cancerous oral mucosae. Spectra were recorded from 27 independent models and subjected to LDA. Data were grouped as normal versus cancer (sensitivity of 100% and specificity of 70%), normal versus dysplastic versus cancer (dysplastic and cancer spectra were classified with a sensitivity of 90 and 98%, respectively, and a 75% specificity) and dysplastic versus cancer (90% sensitivity) (Ref. 39).

Ibrahim *et al*. (2021) probed epithelial and connective tissue compartments using RS to differentiate between benign; dysplastic (mild, moderate and severe) and malignant lesions (OSCC) (Ref. 40). Within the epithelium, as disease severity increased, nucleic acid contributions increased whereas those of protein and lipids decreased. When the connective tissue was probed, nucleic acid peaks became more prominent with disease progression whereas the opposite was true for collagen peaks. When partial least squares discriminant analysis (PLS-DA) classification and leave-one-patient-out-cross-validation was performed for epithelial tissue, the accuracy was highest for OSCC, intermediate for benign lesions, moderate and severe dysplasia and lowest for mild dysplasia. Connective tissue associated with OSCC could be classified with high sensitivity (88%) and specificity (72%) (Ref. 40).

The intensity of 12 Raman bands corresponding to either L-tryptophan or keratin changed noticeably upon transition from normal through carcinoma in situ to invasive OSCC. The results suggested that the enhancement of tryptophan and down-regulation of keratin may be linked to the progression of lingual OSCC, achieving classification accuracies of 89.2, 85.5 and 84.9% for normal, in situ and invasive OSCC, respectively (Ref. 41). This study further highlights the importance of keratin for characterising OSCC and the potential of RS to monitor any changes associated with it.

A SERS catheter tool which demonstrated proof-of-concept for potential real-time detection, classification and grading of ex vivo premalignant and malignant tissue has been developed (Ref. 42). Spectra were recorded from OSCC, verrucous carcinoma, leukoplakia and normal oral tissue, and classified using PCA-DA cross-validation. Normal and leukoplakia spectra were classified with an overall accuracy of 100%; OSCC and verrucous carcinoma spectra were classified with an accuracy of 94.29 and 94.67%, respectively. This catheter-based system combined with multivariate analysis, achieved an overall accuracy of 97.84% for their grading-based spectral classification of OSCC (Ref. 42).

The grading systems of OED present a wide variation in their predictive value. A study was undertaken to evaluate The World Health Organisation's classification system for grading dysplasia using RS to probe for biochemical differences. Although spectral variation was observed between normal mucosa, OSCC and mild, moderate and severe dysplasia (particularly between peaks associated with DNA and proteins), there were no significant differences amongst the dysplastic grades. Moreover, SVM classification of spectra yielded accurate results between OSCC and mild and severe dysplasia, but performance was poor for moderate dysplasia (Ref. 43). Overall, these findings suggest that the incorporation of biochemical constituents to OED grading, as opposed to solely relying on architectural and cytological manifestations, could afford improvements to classification.

### Ex vivo studies of liquid biopsies

There has been a growing interest in liquid biopsies as they allow rapid, real-time and non-invasive analysis with an ease of resampling for assessing the lesion as it evolves. Blood, plasma, serum, exfoliated cells, saliva and urine have been studied in oral cancer using RS.

#### Blood, plasma and serum

Blood constituents are known to reflect changes in the body associated with physiological or pathological states and there is a growing interest in the use of blood-based biomarkers for cancer detection. These are minimally invasive and can provide an accurate representation of the tumour dynamics during disease progression or following treatment. As RS can simultaneously probe the unique signatures of numerous biomolecules in blood, it is well suited for rapid evaluation of the presence of cancer or premalignant conditions. Therefore, there has been a lot of interest in the use of RS for the detection of different biomarkers in whole blood, serum or plasma for breast (Ref. 44), lung (Ref. 45), ovarian (Ref. 46) and head and neck cancers (Ref. 47).

Serum is the most common blood-based medium to which RS has applications for the identification of spectral biomarkers. However, there have been a small number of applications for the characterisation of blood plasma (Ref. 48) and whole blood (Ref. 33). Rekha *et al*. (2013) explored the use of RS to investigate biomolecules present in plasma of clinically confirmed OSMF lesions, one of the OPMDs. In comparison to normal, plasma from OSMF exhibited significant spectral variations with regards to intensity, peak position and peak presence/absence. Moreover, the RS of OSMF plasma had characteristically intense bands for amino acids and proteins compared to normal and malignant samples and PCA-LDA and LOOCV classification accuracy was 84.2% for distinguishing OSMF plasma from normal (Ref. 48). Using whole blood, normal subjects and those with premalignant and malignant oral lesions could be discriminated with 78% accuracy (Ref. 49).

Both conventional and surface-enhanced Raman could classify serum from oral cancer patients and healthy subjects with ~78% classification efficiency and diagnostic accuracy of 81.1% (Refs 50–52). Moreover, spectral differences have been observed between serum from patients with different subtypes of oral cancer (OSCC versus mucoepidermoid carcinoma) (Ref. 51) and between subsites (buccal versus lingual) (Refs 50, 52). Spectra were collected from serum samples from normal, disease control (cancers other than oral), oral premalignant and oral cancer subjects. Variations related to amino acids, Amide III and DNA peaks were noted across different groups. A four-group model approach achieved an 85% sensitivity and 90% specificity for normal versus oral cancer, as well as identifying premalignant and non-oral cancer disease controls with an efficiency of 71 and 66%, respectively (Ref. 53).

Another study used gold nanoparticle-based SERS to preoperatively diagnose and stratify tumour sizes, lymph node involvement stages and pathologic status of OSCC patients from serum samples. With regards to tumour size, the SERS spectra were classified into four groups as T1, T2, T3 and T4 and these were discriminated with a total accuracy of 76.3% using PCA-LDA. In terms of lymph node involvement, patients were grouped into N0, N1 and N2 and a total accuracy of 85.9% was achieved using PCA-LDA. Finally, PCA-LDA achieved a total accuracy of 90.4% for discriminating grade I, II and III OSCC (Ref. 54).

These studies suggest the potential of serum-based RS as a simple and minimally invasive screening tool for the detection of disease progression and lymph node metastasis in oral cancer.

Although rare, some primary cancers can metastasise to the oral cavity (Ref. 55). Therefore, establishing whether the oral cavity is the primary or secondary site of a tumour is important. SERS spectra derived from OSCC patient serum could be distinguished from those from four different primary solid malignancies: breast, lung, colorectal and ovarian. Oral cancer samples were differentially classified with the highest accuracy, achieving 88% (PCA-LDA) (Ref. 56). Further, RS could distinguish the serum of primary glioma and oral cancer patients with 89% efficiency (Ref. 53).

Oral cancer recurrence was identified with ~80% efficiency by serum RS analysis of post-surgery samples; recurrent and non-recurrent oral cancers could not be differentiated from pre-surgery serum (Ref. 57). In a recent publication (Ref. 58), it was shown that biochemical differences related to disease recurrence could not be detected in serum acquired before surgery. However, PC-LDA could classify recurrent and non-recurrent serum with an 81% specificity and 86% sensitivity in post-surgery serum. Interestingly, even serum collected as early as 1-week post-surgery could accurately identify subjects at highest risk (Ref. 58).

#### Exfoliated cells and saliva

Saliva is composed of water, inorganic and organic substances, proteins, glycoproteins and exfoliated cells. In the past decade, salivary biomarkers gained recognition in molecular diagnostics (Ref. 59). Exfoliative cytology and saliva sampling offers several advantages such as its minimally invasive nature, feasibility for resampling and it also offers a practical approach for monitoring patients where there are limited resources.

*Exfoliated cells:* Traditional exfoliative cytology is based on microscopic examination of cell smears. This suffers from low sensitivity for diagnosing oral cancers, which is primarily attributed to subjective interpretation of microscopic findings (Ref. 60). An initial exploratory study confirmed the feasibility of acquiring Raman spectra from exfoliated cells and demonstrated a preliminary classification of premalignant and healthy patients. Exfoliated cells from the buccal mucosae from sites of increasing disease severity (healthy, high-risk chronic tobacco users and the contralateral normal and tumour sites of OSCC patients) were assessed. Upon increased disease severity, there was an increase in intensities of DNA (1095 and 1325–1330 cm^−1^), CH_2_ bending (1450 cm^−1^) and Amide III bands, as well as a broadening of the Amide I region (Ref. 61). Raman-based exfoliative cytology has subsequently demonstrated continued efficacy for distinguishing healthy subjects from patients with OPMDs with high sensitivity and specificity (Refs 62–65). Moreover, spectral differences between desquamated oral cells from healthy controls and OSCC patients have also been observed. The 1347 and 1543 cm^−1^ bands were more prominent in OSCC spectra, whereas the 1409 cm^−1^ band was completely absent. PCA-LDA and LOOCV analysis revealed a good classification with sensitivity and specificity of 68 and 52% (Ref. 66).

Using an integrated Fourier-transform infrared Raman PCA-LDA model to analyse exfoliated cells, classification accuracies of 97.7 and 86.7% in spectrum- and patient-wise approaches were achieved for discriminating normal, premalignant and cancerous conditions. This dual spectral approach also identified Raman signatures of cancer progression with alterations to protein secondary structure, high CH_2_ bending and broad amide I and III regions from healthy volunteers to cancer patients. Moreover, the spectra were averaged and subject to ‘difference between mean spectra’ analysis and several peaks between 831 and 1346 cm^−1^ discriminated between the three groups (Ref. 67).

In a more recent study, the discriminatory power of a machine learning ensemble classification, consisting of an LDA-SVM algorithm for distinguishing normal, premalignant and malignant on Raman exfoliated cyto-spectral data, was demonstrated with an 88% accuracy. The risk of oral cancer in susceptible populations (i.e. tobacco users) was also evaluated by applying a machine learning model trained on spectra from cigarette smokers and healthy controls. The similarity of the spectra from cigarette smokers with spectra from precancer and cancer lesions highlighted the potential of their LDA-SVM model for risk assessment (Ref. 68). Indeed, the prognostic prediction potential of cyto-RS in oral cancers has been corroborated by another group (Ref. 69). Exfoliated Raman cytology has also been shown to accurately classify high- and low-grade dysplasia (Ref. 63) and detect field cancerisation effects (Ref. 64).

*Saliva:* Studies have demonstrated the potential of using saliva to monitor a range of physiological and pathological changes. Subsequently, saliva has been studied as a source of biomarkers in the diagnosis of cancers, particularly oral cancers, as this biofluid is in the closest proximity to the oral cavity and may contain metabolites lavaged directly from tumours (Refs 70, 71). Conventional spontaneous and surfaced-enhanced Raman methods have been employed to distinguish saliva samples (fasting, unstimulated) from healthy and OSCC patients. Following centrifugation and lyophilisation, saliva samples were probed with conventional RS and following PCA-LDA analysis, spectra were classified with 93.6% sensitivity, 94% specificity and 94% diagnostic accuracy (Ref. 72). SERS spectra were acquired from purified saliva (fasting, unstimulated) of five healthy and five OSCC patients. This revealed Raman bands at 1097 and 1627 cm^−1^ in a proportion of cancer samples that were otherwise absent in normal spectra (Ref. 73). This was later corroborated when both silver and gold nanoparticle-based SERS analyses of saliva followed by multivariate statistical approaches could differentiate cancer patients from healthy subjects (Refs 66, 74). SERS has also demonstrated the ability for the rapid detection of an established and reliable biomarker of OSCC, known as S100 calcium-binding protein P (S100P) mRNA using saliva samples (fasting, unstimulated). Gold nanoparticles were used as the SERS substrate and DNA oligonucleotides were conjugated to recognise S100P mRNA from saliva samples. The average concentration of S100P mRNA was three times greater in the OSCC patients (Ref. 75). This work highlights the potential for SERS-based salivary biomarker detection for rapid OSCC diagnosis.

RS has the potential for objectively discriminating saliva from normal, premalignant and malignant samples (Refs 33, 76). Spectra from saliva from healthy subjects or patients with OSMF or OSCC were subject to PCA-LDA. Healthy and premalignant unstimulated saliva could be differentiated with a sensitivity of 96.4 and 93.8% healthy versus malignant unstimulated saliva could be classified with a specificity of 70.2 and 95.7%, respectively (Ref. 76). A similar approach distinguished normal, premalignant and malignant groups with a classification efficiency of 91.3%, with peaks corresponding to oxygenated haemocyanin (752 cm^−1^), carotenoids (1158 and 1525 cm^−1^) and mucin matrices (1444 cm^−1^) indicating demonstratable variation between different classes. The highest accuracy for discriminating OPMDs or OSCC from normal subjects was using unstimulated saliva compared to urine and blood (Ref. 33). Moreover, Raman and SERS signatures of saliva combined with PLSDA could also discriminate between healthy subjects and those with mild and moderate OED (Ref. 77).

#### Urine

Urine is considered a medium of significant diagnostic potential in a variety of metabolic and pathological conditions. More recently, attention has turned to its applicability in cancer detection and a number of studies used RS to characterise urinary metabolites in breast (Ref. 78), cervical (Ref. 79), prostate (Ref. 80) and bladder cancer cases (Ref. 81).

For oral cancer and OPMDs, a study focused on characterising urine taken from healthy subjects and oral cancer patients. Using a confocal micro-Raman system revealed several biochemical differences between the two groups including the intensity of peaks. These correspond to bond vibrations of urinary metabolites such as uric acid, creatine, DNA, indoxyl sulphate, amino acids, pteridine, neopterin and flavins as well as a blue shift of the 1002 cm^−1^ band associated with the phenylalanine vibrational mode. PCA-based LDA and LOOCV could identify cancer patients from normal subjects with a 98.6% sensitivity, 87.1% specificity and 93.7% accuracy (Ref. 82). In a study by the same group, PCA-LDA and LOOCV of urine spectra from healthy and patients with premalignant lesions yielded 86.3, 92.9, 90.9% sensitivity, specificity and overall accuracy, respectively (Ref. 83).

By studying the high-wavenumber region of the Raman spectra from urine samples, distinct differences were observed in bond vibrations associated with flavins, tryptophan and phenylalanine between OSCC, OPMD and healthy controls (Ref. 84). A PCA-LDA and LOOCV diagnostic algorithm established a specificity of 98.7% in detecting normal subjects; and sensitivity of 91.9 and 86.6% for detecting OPMDs and oral malignancy, respectively. Analysing (PCA-LDA) the fingerprint region of the spectrum from urine samples taken from OSCC, OPMDs and healthy subjects revealed a classification accuracy of 90.5% (Ref. 33).

### In vivo studies of oral tissues

The oral cavity is one of the most accessible regions of the body. In previous sections, we have highlighted the potential for RS to detect, characterise and diagnose oral cancer and OPMDs ex vivo, including tissue and liquid biopsies. This promising work is a proof-of-principle, and the obvious next steps are to explore the potential of non-invasive in vivo Raman systems to characterise, detect and diagnose oral cancer and OPMDs and transfer this technology from benchtop to chairside.

The first studies that recorded in vivo RS measurements from the oral cavity focused on exploring its feasibility in acquiring spectra and investigated its discriminatory power between different anatomical locations (Refs 85, 86). Early studies that employed in vivo RS measurements to study oral tissue recorded in vivo spectra at different points in the buccal mucosa of healthy volunteers in short and clinically acceptable time (Ref. 87). Recent work has been carried out to characterise biochemical features of various oral subsites (lip, tongue, gingiva and buccal mucosa) in vivo from healthy subjects. Average spectra from each subsite were subject to spectral deconvolution and curve fitting to separate contributions from different biochemicals (Ref. 88). The differences observed between subsites can be used to improve contrast against pathological conditions, particularly for detecting precancerous changes. Moreover, sub-site classification also improved overall in vivo classification of normal, malignant and OPMDs in another study (Ref. 89).

A classification efficiency of 82.4% for normal and 89.9% for cancerous mucosae was achieved through the application of intra-oral Raman probe combined with LDA and LOOCV (Ref. 90), and demonstrated the potential of in vivo classification of premalignant and malignant oral lesions (Refs 91, 92). Normal, premalignant and cancerous lesions of the buccal mucosa were probed and biochemical differences between the spectra, similar to those identified by other groups on ex vivo samples, were highlighted including in Amide I and II peaks.

Differences in the spectra were further explored by LDA, followed by LOOCV and 71.3% of normal mucosa, 77.6% of tumour and 50.6% premalignant lesions were correctly predicted (Ref. 91). In similar studies, in vivo spectra were recorded from the mucosa of patients previously diagnosed with benign hyperkeratosis, inflammation, mild, moderate and severe dysplasia or OSCC. Spectra were split into two groups: the first containing spectra from normal mucosa and benign lesions, and the second from dysplastic lesions and OSCC. The analysis was able to discriminate between these groups with 100% sensitivity and 77% specificity. As the sample size was limited, individual comparisons between the subgroups were not possible (Refs 92, 93).

RS was employed to explore the sequence of biochemical alterations that accompany oral carcinogenesis using an experimental animal model that progresses through hyperplasia, dysplasia, carcinoma in situ and OSCC, as per human disease. In vivo Raman spectra were acquired from the buccal pouches of carcinogen-treated hamsters every week for 14 weeks post-treatment. Lipids dominated the spectra in the early stages, whilst in the later stages, tumours showed an increased protein-lipid ratio and nucleic acids. Moreover, in an ex vivo arm of the study animals were sacrificed each week over the 14-week, and the buccal pouches excised for analysis. Similar results were found in terms of spectral signatures and trends of PCA classification, demonstrating the translational potential of RS for monitoring oral cancer progression (Ref. 94).

The utility of in vivo RS was demonstrated for detecting subtle changes associated with early neoplastic transformation in uninvolved areas of the human buccal mucosa exposed to tobacco (Ref. 95). The same group assessed the potential of RS in predicting second primary tumours and local recurrence in patients with OSCC by screening the mucosa contralateral to the primary tumour. They hypothesised that misclassification of normal tissue was an early indicator of underlying carcinogenic events. Correlations between misclassifications of normal mucosa and the appearance of any new malignant lesions in the oral cavity were investigated. Patients whose normal spectra mismatched with that of tumour had a 1.5x higher chance of developing local recurrence (Ref. 96). These findings confirm the ability of RS to assess the impact of field cancerisation and to predict new or recurrent tumour formation.

The potential utility of in vivo RS to predict disease-free survival (DFS) in OSCC patients has been explored (Ref. 97). In vivo spectra were acquired from patients with OSCC, both from the tumoural zone and contralateral normal mucosa, using a portable Raman system coupled to a fibre-optic probe. These patients then underwent surgery and adjuvant therapy, when appropriate, and the duration of DFS was measured from this date to that of a recurrence; if no recurrence had occurred these patients were considered disease free at this follow-up date. Survival models and Cox proportional hazard analysis were performed and 76 and 104 Raman spectral points, between 1200 and 1800 cm^−1^, of the tumour and contralateral normal tissue could accurately predict DFS. The three highest ranked Raman shifts (1365.30, 1351.84 and 1488.70 cm^−1^), as determined by the smallest generated *P*-values, were selected from the tumour spectra to illustrate their correlation with DFS. These peaks were assigned to the vibrational modes of the purine DNA bases and thus differential DNA content appears to have a significantly higher power for predicting OSCC disease recurrence (Ref. 97).

## Perspective: opportunities, strategies and challenges for clinical translation

Histopathology remains the gold-standard for diagnosing oral cancer and OED.

Ex vivo Raman analysis of histopathological biopsies can probe biochemical changes and offer insights into disease progression and treatment response thus complementing traditional histopathological assessment. Findings from the studies summarised in the previous section show great promise in the use of RS on ex vivo solid samples with most of the studies showing excellent sensitivity and specificity in detecting cancerous from normal tissue. RS also showed promise in identifying premalignant tissues but with lower accuracy. This is expected given the known lack of reproducibility and subjectivity in the classification of OED, which is one of the most problematic areas of pathology (Ref. 98). Therefore, technologies like RS in combination with digital pathology can help improve these grading systems by identifying specific molecular and chemical changes associated with the changes in morphometry seen histopathologically. Indeed, changes in the RS bands representing lipids, carbohydrates, proteins, amino acids and nuclei acids were demonstrated in premalignant and malignant oral tissues. The shift toward protein signatures in malignancy coupled with a reduction in lipid content, in addition to changes in cytokeratins and water concentration, should be explored further for identifying early markers of disease progression. These need to be coupled with changes in architecture at the tissue and cell level observed microscopically which can be assessed objectively using a plethora of techniques such as digital pathology, machine and deep learning (Ref. 99).

Interestingly, some studies could identify differences between normal and malignant tissues in the epithelial compartment and not the sub-epithelial tissues. This is worth exploring further as morphologically, differences have been observed in the sub-epithelial connective tissue, including changes in the distribution of collagen IV which we had previously reported (Ref. 100).

RS also identified differences in the chemical fingerprint of different sites in the oral cavity. This aligns with the known architectural differences (Ref. 101), in addition to the different prognosis for OSCC depending on the lesion site. This requires further assessment to identify site-specific markers of disease progression.

Currently, oral cancer screening is performed visually by dental practitioners during routine dental check-ups. Saliva RS-based assays and in vivo RS using hand-held intra-oral probes could complement this by providing non-invasive, quantitative and rapid assessment of the oral mucosa to aid in the detection of precancerous and cancerous lesions. RS could provide great benefit during the earliest stages of the disease when lesions are difficult to detect visually. This is especially important to pursue given accessibility of the oral cavity and the non-invasive nature of such screening tests.

Other non-invasive, easily accessible samples include ex vivo liquid samples, the RS analysis of which showed reasonable differentiation between oral cancer and healthy subjects. While serum samples did not offer the same discrimination power between health and disease compared to biopsies, much better results were observed with saliva samples. This is expected given the proximity to the diseased tissue. Given the ease of sample acquisition for screening and follow up, further work is needed to identify targeted biomarkers to develop portable screening tests from blood and saliva that can be widely used in different settings and analysed using RS. With the introduction of portable handheld RS platforms, including those with software control via mobile phones, community screening is within touching distance.

Findings from ex vivo studies can also inform the development of targeted clinical handheld RS systems that can be used for chairside screening and for guiding in vivo surgical procedures. There are a number of light-based diagnostic techniques under study. One method is narrow band imaging (Refs 102, 103) that can detect changes in submucosal vasculature, and another is diffuse reflective imaging that uses spectral imaging to discriminate healthy from premalignant and malignant tissues (Ref. 104). Methods that aim at enhancing the visibility of the oral lesions also include Vizilite, a tissue reflectance-based test (Ref. 105) and various fluorescence molecular imaging techniques (Ref. 106) including auto-fluorescence imaging using Velscope (Ref. 107). These techniques have great promise for chairside screening as they have shown good sensitivity in detecting OSCC. However, many still suffer poor specificity, which is problematic as false-positive diagnosis could lead to unnecessary invasive treatment and increased patient anxiety. Furthermore, some of these technologies only focus on one aspect of OSCC pathogenesis (e.g. narrow band imaging only visualises changes in vasculature) and some are not cost effective as screening tests. Introducing RS into clinical practice has the added advantage of targeted screening based on identified biomarkers, and the potential of expansion to the full chemical fingerprint. There is potential for combining some of the existing light-based technologies with RS to achieve more accurate detection of even the earliest changes. This is also important during surgical treatment of identified lesions, as achieving adequate resection margins is crucial for improving patient prognosis, and currently, these are determined intraoperatively and confirmed by histological examination of frozen sections. On its own or in combination with other light-based technologies, in vivo RS has the potential for objective intra-operative definition of surgical margins in real-time. Identification of early biochemical changes using RS intraoperatively could help surgeons delineate tumour boundaries accurately to ensure complete excision.

With regards to RS data analysis, machine and deep learning need to be further employed to detect changes in different tissues. Most of the reported studies employed variations of PCA for spectral analysis. While effective, important information could be missed using PCA leaving room for other analytical methods to complement existing ones.

Despite the advantages of RS in examining various types of samples and providing biomolecular information, it suffers from shortcomings. Raman scattering is a weak signal and longer acquisition times are required to compensate for this weakness. Furthermore, autofluorescence can contaminate the biological fingerprint of the tissue and further dampen the signal. The combination of RS with other optical techniques such as infrared spectroscopy can overcome these disadvantages (Refs 108–112).

Whilst RS shows great promise, further validation and standardisation is imperative before it can transition from benchtop to chairside clinical applications. The acquisition and interpretation of Raman data is subject to significant variation from instrumentation and analysis techniques. There is a need to standardise the acquisition and analysis of biologically meaningful spectra. Robust validation studies will be necessary to ensure centre–centre reproducibility using optimal acquisition parameters. The interpretation of spectra requires molecular biology and vibrational spectroscopy expertise, which may not be currently available to healthcare professionals. The coupling of RS with multivariate statistical approaches facilitates the transformation of highly complex data into manageable variables that confer the underlying biological mechanisms. Overall, additional training for acquiring and interpretating spectral data is necessary to facilitate the integration of RS into clinical decision making. This could be facilitated by the development of automatic and user-friendly software to increase the accessibility of RS-based analysis without disrupting workflow efficiency.

With the promising preliminary results from in vivo RS work in oral applications, continued development of fibre-optic probes and portable handheld devices is necessary to bring RS into a clinical setting. Compared to benchtop systems, these are considerably less expensive, require less space and most importantly, can be used intra-operatively. However, to date, no large-scale in vivo clinical studies have been conducted for oral cancer and OPMD diagnosis. Validation in large-scale clinical trials is imperative to demonstrate its safety, efficacy and cost-effectiveness.

With the advances in RS technologies such as spatially offset Raman spectroscopy, transmission Raman spectroscopy and shell-isolated nanoparticle-enhanced Raman spectroscopy, in addition to novel data acquisition and processing using artificial intelligence and machine learning, the potential of applying RS into the study of oral cancer and OPMDs is constantly expanding.

Given the constant development of RS hardware and software, coupled with advances in data interpretation, there is great scope for combining RS with other technologies such as digital pathology, machine learning and Fourier transfer infra-red spectroscopy in the study of oral cancer. The ultimate aim would be incorporating these state-of-the-art technologies into the existing clinical workflow to improve patient outcome.

**Figure 3.**
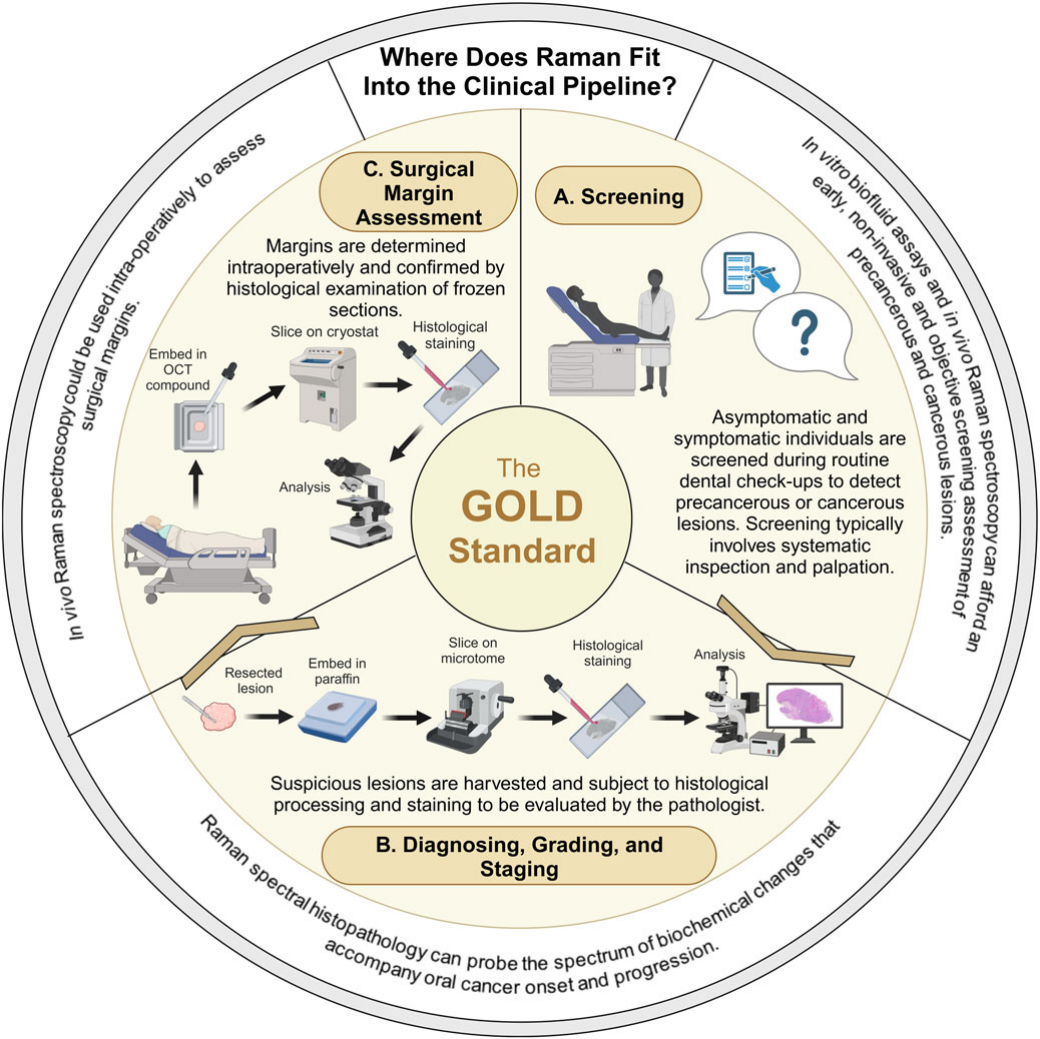
Raman spectroscopy could complement the pipeline within clinical practice for oral cancer and OPMD detection, diagnosis and surgical margin assessment. (A) The current standard for screening is via conventional visual oral examination under a bright light source to detect abnormal oral findings and involves systematic inspection and palpation of the oral cavity and regional lymph nodes. In vitro (liquid biopsies) and in vivo (hand-held) Raman spectroscopy have demonstrated efficacy to assist with conventional screening, objectively, rapidly and at low-cost. (B) To achieve a definitive diagnosis, a tissue biopsy followed by histological assessment is the current gold-standard for oral cancer and OPMD diagnosis. This is invasive and prone to interpretative disparity amongst pathologists. Raman spectroscopic evaluation of small ex vivo tissue samples or thick sections has demonstrated potential to assist in the diagnosis and prognosis prediction of oral cancer and OPMDs. (C) When oral cancer is diagnosed, surgery is the main treatment and achieving adequate resection margins (greater than 5 mm of surrounding healthy tissue) is imperative for improving the prognosis intraoperative assessment can drastically improve the assessment of tumour resection margins. Currently, this is labour intensive, time-consuming and subjective. In vivo Raman spectroscopy affords potential as an objective and easy-to-use technology, which could be used intra-operatively to accurately demarcate surgical margins. Created in Biorender.

Overall, this review has demonstrated that leveraging the strengths of RS alongside conventional techniques can enhance the accuracy and efficiency of the detection, diagnosis and management of oral cancer and OPMDs (Fig. 3).

## Summary

Oral cancer survival is not improving; hence, there is an urgent need to develop highly sensitive and specific tools that can assist in early detection of disease progression. RS is a label-free analytical tool that can objectively probe the biochemical changes that accompany premalignancy and oral cancer onset. By investigating structural and compositional molecular changes in oral tissue, RS has demonstrated efficacy in distinguishing normal, premalignant and malignant oral samples ex vivo and has shown promise in early in vivo studies. Moreover, RS has demonstrated potential in characterising oral tumours and OPMDs, where spectral signatures can inform lesion classification, grading and risk prediction. The emerging interest of RS for characterising liquid biopsies, particularly saliva, which is easily accessible, and in direct contact with the oral tissues, could be transformative for real-time monitoring of at-risk individuals and serve as a simple non-invasive screening and diagnostic tool to guide OPMD and OSCC patient management. Improvements to existing and newly emergent Raman technologies, as well as novel methods for analysing Raman data, in combination with other technologies, such as digital pathology and artificial intelligence, are exponentially improving its sensitivity, specificity and accuracy for applications in oral cancer and OPMDs.

## Supporting information

Hanna et al. supplementary material Hanna et al. supplementary material

## Supplementary material

For supplementary material accompanying this paper visit http://doi.org/10.1017/erm.2024.26. click here to view supplementary material
