# Supplementary material for: Advances in Raman spectroscopy for characterising oral cancer and oral potentially malignant disorders
Source: Expert Rev Mol Med. 2024 Oct 8;26:e25. doi: 10.1017/erm.2024.26 (PMC11488342; doi:10.1017/erm.2024.26)
Supplement: Hanna et al. supplementary material [file S1462399424000267sup001.docx]

**Supplementary file**

Raman variants

Spontaneous Raman scattering is an inherently weak process; only one in a million photons are scattered inelastically. Surface enhanced Raman spectroscopy (SERS) affords a large signal intensity enhancement through the use of a roughened metallic surface and thus facilitates the detection of molecules only present at very low concentrations. SERS is therefore particularly beneficial for the analysis of liquid biopsies, which contain trace levels of biomolecules. Shifted-excitation Raman difference spectroscopy (SERDS) is another variant that was first proposed to overcome strong autofluorescence interference. Here, two very similar wavelengths of light are chosen to excite the sample under investigation; the small change in wavelength induces a shift in the Raman spectrum whilst the fluorescence signal remains unchanged. The two spectra are subtracted from each other to produce the Raman difference spectrum, which is free from fluorescence signals. This technique is particularly beneficial for the analysis of fresh biological tissue.

Statistical approaches for analysing Raman spectra

The coupling of Raman spectroscopy with statistics enables the generation of quantitative spectral-based biomarkers. Univariate statistical approaches involves a simple approach where individual features of Raman spectra are extracted, including band positions and intensities, and evaluated for their use as a means of disease classification. The second type of approach, which is arguably more common, is based on multivariate statistics. Multivariate approaches are more computationally demanding and complicated, and algorithms are implemented to recognise subtle differences in vibrational signatures between different samples. Machine learning facilitates the identification and emergence of trends and patterns, within data, to establish mathematical models on a training dataset, which are subsequently used as a predictor for similar, unseen test data.

Pattern recognition can broadly be classified as either unsupervised or supervised, with the former often used as a precursor to supervised methods for the analysis of very large datasets. Unsupervised methods serve to reduce the dimensionality of the highly complex data whilst not requiring any prior knowledge of the sample. It is concerned with identifying hidden patterns in the data and therefore exploratory in nature. This type of method is often referred to as cluster analysis and some of the most commonly applied algorithms include, principal component analysis (PCA), k-means clustering and hierarchical clustering analysis. Unlike unsupervised methods, supervised methods rely on pre-existing class labels, such as the histopathological diagnosis, to obtain an accurate predictive model. Supervised methods include, linear or quadratic discriminant analysis (LDA or QDA), partial least squares discriminant analysis (PLSDA), convolutional neural networks and support vector machines (SVM).
